# The association between exaggeration in health-related science news and academic press releases: a replication study

**DOI:** 10.12688/wellcomeopenres.15486.2

**Published:** 2019-11-18

**Authors:** Luke Bratton, Rachel C. Adams, Aimée Challenger, Jacky Boivin, Lewis Bott, Christopher D. Chambers, Petroc Sumner

**Affiliations:** 1 School of Psychology, Cardiff University, Cardiff, Cardiff, CF10 3AT, UK; 2 Cardiff University Brain Research Imaging Centre (CUBRIC), School of Psychology, Cardiff University, Cardiff, Cardiff, CF10 3AT, UK

**Keywords:** hype, media, science communication

## Abstract

**Background:** Exaggerations in health news were previously found to strongly associate with similar exaggerations in press releases. Moreover, such press release exaggerations did not appear to attract more news.

**Methods:** Here we tested the replicability of these findings in a new cohort of news and press releases based on research in UK universities in 2014 and 2015. Press releases and news were compared to their associated peer-reviewed articles to define exaggeration in advice, causal claims and human inference from non-human studies.

**Results:** We found that the association between news and press releases did not replicate for advice exaggeration, while this association did replicate for causal claims and human inference from non-human studies. There was no evidence for higher news uptake for exaggerated press releases, consistent with previous results. Base exaggeration rates were lower for human inference from non-human studies, possibly reflecting the Concordat on Openness on Animal Research in the UK.

**Conclusions:** Overall, the picture remains that the strength of news statements is normally associated with the strength of press release statements, and without evidence that exaggerated statements get significantly more news.

## Introduction

Established news media are a widespread means to disseminate beneficial health information to millions of readers. However, simplifying complex scientific findings into appealing news stories often creates misleading claims ( Haneef *et al*., 2015; Yavchitz *et al*., 2012) that could potentially damage public health and create confusion and mistrust ( Grilli *et al*., 2002; Matthews *et al*., 2016; Ramsay, 2013; Schwitzer, 2008; Sharma *et al*., 2003). The Academy of Medical Sciences recently reported that only 37% of British adults generally trust scientific evidence ( The Academy of Medical Sciences, 2017).

Science and health news is often stimulated by press releases from journals, universities and funders ( Autzen, 2014; de Semir *et al*., 1998). Previous observational research has found that health news content is strongly associated with press release content ( Jackson & Moloney, 2015; Lewis *et al*., 2008; Schwartz *et al*., 2012; Sumner *et al*., 2016; Sumner *et al*., 2014; Yavchitz *et al*., 2012). In a previous study, we analysed three types of common subtle exaggeration: advice given to readers, causal claims based on observational data and claims about humans based on non-human research ( Sumner *et al*., 2014). We based the study on health-related press releases from major UK universities in 2011. We found that a third or more press releases contained stronger advice, causal statements or human claims than we found in the peer-reviewed journal article they were based on (this was our operating definition of exaggeration). These subtle exaggerations in press releases strongly predicted their presence within news. However, exaggerated press releases were not more likely to attract news, counter to the common assumption that hyping scientific findings should help in getting more news interest.

The study by Sumner *et al.* (2014) attracted interest and controversy, and also formed part of the basis for the Academy of Medical Sciences’ recommendations for improving health news ( The Academy of Medical Sciences, 2017). There have been four partial replications. We found broadly similar results for journal press releases rather than university press releases ( Sumner *et al*., 2016). A similar pattern emerged again recently in a trial of journal and university press releases that concentrated on the issues of correlation and causality in headlines, statements and caveats ( Adams *et al*., 2019). A study of causal claims in German news ( Buhse *et al*., 2018) found similar rates of exaggerated headlines compared to the journal article conclusions (and did not analyse association with press releases in the same way). But the most direct replication for university press releases was by Schat *et al*. ( Schat *et al*., 2018), who investigated 129 health-related press releases from Dutch Universities and 185 associated news articles collected in 2015. The relationship between exaggeration in press releases and exaggeration in news was strongly present. However, contrary to the findings of Sumner *et al*. (2014), who sampled press releases in 2011, Schat *et al.* (2018) found that press releases with exaggeration were 1.5 times (95% CI: 1.02-2.04) more likely to be picked up by the news than press releases that did not contain exaggeration. It is not known whether the discrepancy is due to differences between the countries sampled, changes in broad practice between 2011 and 2015 as the competitive nature of news evolves, or other factors.

Sumner *et al*. (2014) tested three types of exaggeration: causal claims, advice and human claims from animal experiments. For the latter, at least, there is a clear reason to expect a difference between 2011 and 2014/15. One motivation for expressing animal research in human terms would have been to avoid revealing the presence of animal research facilities. Since that time, the majority of the institutions in the sample were involved in the Declaration on Openness on Animal Research (2012) and the subsequent Concordat on Openness on Animal Research in the UK (2014). The institutions committed to “include information about that animal research in relevant communications, including media releases” (Concordat on Openness on Animal Research in the UK, 2014). Indeed, in the recent experimental trial on causal claims in press releases and news, subsidiary analysis of exaggerations for animal studies indicated that such animal-to-human exaggerations seem to have become much rarer ( Adams *et al*., 2019, supplementary information).

The present study tested whether the findings of Sumner *et al*. (2014), would replicate in news arising from UK university press releases in 2014 and 2015, using slightly improved methods (more even sampling and 100% double coding).

## Methods

We followed the procedures of Sumner *et al*. (2014) as closely as possible, except where explicitly stated below.

### Collection of press releases, journal articles and news

Press releases from 2014 and 2015 were collected from the 20 universities who were members of the Russell Group in 2011, the same sample institutions as in Sumner *et al.* (2014). The Russell Group is a group of 24 research-intensive institutions in the UK. For example, in the 2014 Research Excellence Framework, the Russell Group accounted for 68% of all four-star rated research in the country ( Russell Group, 2014). The four institutions that joined the Russell Group in 2012 have been excluded from this replication.

**Figure 1.**
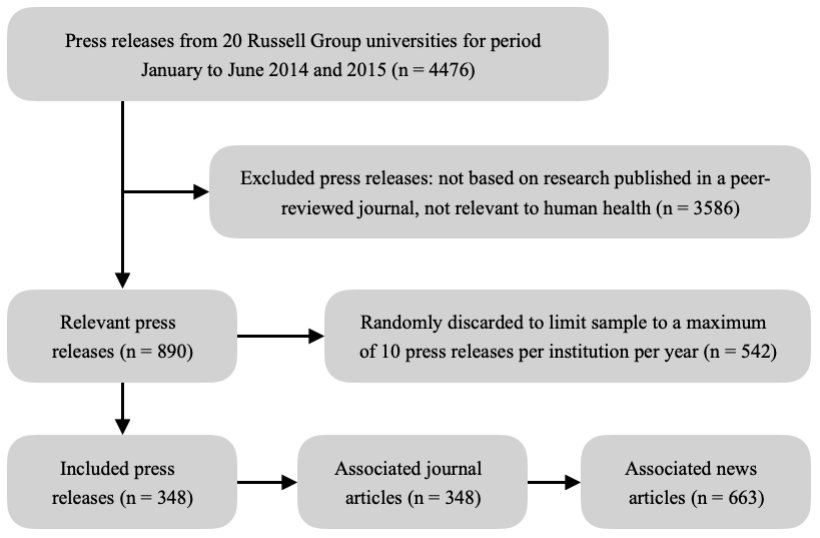
Press release and news selection.

The sample period was January to June 2014, and January to June 2015, in order to sample over two years with a tractable number of press releases. Online repositories (the universities’ websites, and EurekAlert.org) were searched for any press releases from the included institutions. This resulted in a corpus of 4476 press releases. The number of available press releases varied from 90 to 517 across institutions. The sample was then restricted to those relevant to human health, broadly defined (including all biomedical, lifestyle, public health and psychological topics), that reported on a single, published, peer-reviewed research article ( Figure 1). This left 890 relevant press releases. In our previous work we accepted all press releases at this stage, but this would have led to uneven numbers across institutions (7 to 111) and an unmanageable workload for the replication. Here, we therefore implemented a cap of 10 press releases for each time period for each institution, leaving up to 20 press releases per institution. Selection, where necessary, was achieved through randomization. This resulted in a sample of 348 press releases.

For each selected press release, relevant news articles (i.e. those which refer to the source research) in English-language top-tier international and national news outlets were collected via keyword searches using Google Search and the Nexis database (LexisNexis, New York, NY), up to 28 days after publication of the press release, and up to one week before (to allow for rare embargo breaches).

### Article coding

Each press release, associated journal article, and any related news articles (herein referred to as article set), were searched for a number of attributes, which were recorded using the coding sheet included in the open data. This coding sheet contains the relevant subset of questions from the coding sheet used by Sumner *et al.* (2014): the presence and strength of advice, the strength of causal/correlational statements, and the type of sample mentioned (human or non-human). Although Sumner *et al.* (2014) used partial double coding and demonstrated with simulations that a 10% rate of disagreement between coders would not influence their conclusions, in the present study we improved the rate of double coding to 100%; i.e. two researchers independently coded every article set. Subsequently the codes were compared electronically for disagreements and the coders met to discuss disagreements and come to a consensus. On the rare occasions a disagreement was not readily resolved, a third researcher decided between the two alternative interpretations.

***Coding of advice.***We replicated the method in Sumner *et al*. (2014). For advice, there were three levels coded (see Table 1). Advice was coded if it appeared anywhere in the title, abstract, or main text of the journal article, and in the titles or main text of press releases and news articles. If multiple pieces of advice appeared in an article, the strongest piece of advice was recorded. News or press releases were deemed to have exaggerated if they contained a higher code than the corresponding peer-reviewed journal article. For example, ‘explicit advice to the reader’ in a press release is deemed stronger than ‘explicit advice, not to the reader’ in a journal article.

**Table 1.** Coding categories for advice, language, and sample. Categories are listed in descending order of strength, with categories higher up the table trumping categories below.

Advice	Language	Sample
Explicit advice to reader	Cause	Human
Explicit advice not to reader	Can cause	Implicitly human
No advice	Conditional cause	Non-human
	Ambiguous	
	Associative	
	Does not cause	
	No cause mentioned	

***Coding of causal and correlational claims.*** We replicated the method in Sumner *et al*. (2014). The strongest claims relating two variables in the study (e.g. a food and a disease) were recorded from the abstracts and discussion sections of journal articles and coded on a seven-point scale ( Table 1). For press releases and news articles, the strongest statement was coded from the first two sentences of main text (where these were directly relevant to the research; occasionally news leads with general context, which was excluded). This strategy was employed because of the inverted pyramid structure of news, where the most pertinent points are presented first. Only article sets where claims were based on correlational data were used for analysis.

***Coding of samples.*** We replicated the method in Sumner *et al*. (2014). Statements about the sample were rated on a three-point scale ( Table 1), and only journal articles with non-human samples were used for the comparison.

### Analysis

Three types of analysis were carried out:

1. quantifying levels of press release exaggeration;

2. association of news exaggeration with press release exaggeration;

3. news uptake.

***Advice.*** To analyse advice, only cases where advice appeared in at least one article in the article set were selected. This means that cases where no advice appeared across the journal article, press release, or news were excluded because exaggeration could not be measured. The sample sizes then became 74 press release/journal article pairs of for analysis of press release exaggeration rates and 70 press releases and journal articles for analysis of associations with news, with 248 associated news articles.

***Causal and correlational claims.*** This analysis only included cases where the design of the study reported in the journal article was observational. Interventions, computer models/simulations, qualitative designs, and meta-analyses were excluded from this analysis. The sample size for this analysis was 154 press releases; 58 of these press releases had news. There were 237 associated news articles.

***Inference from non-human samples.*** Studies reporting on human samples were excluded from these analyses. The sample size for this analysis was 117 press releases based on studies with non-human samples that could be used to calculate press release exaggeration percentages. For the analysis of exaggeration present in the news, there were 38 press releases available with 129 associated news articles.

## Statistical analysis

Consistent with our previous approach ( Sumner *et al*., 2014), generalised estimating equations (GEE) were used (in SPSS version 24) to provide estimates and confidence intervals adjusting for the clustering of multiple articles to one source (multiple news articles from one press release; or multiple press releases from the same institution). The GEE is an extension of the quasi-likelihood approach and is used in situations in which data is clustered to estimate how much each data point should contribute statistically. The key part of the process is to estimate the correlation of data within clusters. At one extreme, all data within clusters might be fully correlated, in which case there is really only as many samples are there are clusters; separating the data points within clusters adds no additional information. At the other end of the extreme, data within clusters may be entirely uncorrelated; in this case the clustering does not matter and all data points can be treated as independent. In reality, data within clusters tends to be somewhat correlated, and the GEE estimates this and applies a weighting factor to those data points depending on the degree of correlation. The approach is accessibly explained by Hanley *et al*. (2003), so we do not replicate the equations here. We used a logit linking function because the data is binary, and an exchangeable working correlation, which is a common approach for clustered data and makes a parsimonious assumption that correlations are similar between all data within clusters.

## Results

### Press release exaggeration

***Advice.*** In total, 51% (95% confidence interval = 40% to 62%) of press releases where advice existed in the article set contained advice not present in the journal article, or advice that was more direct than that in the journal article. Whether or not this should be considered exaggeration is addressed in the *Discussion*.

***Causal claims from correlation.*** In total, 27% (95% confidence interval = 21% to 35%) of press releases about correlational research contained a causal statement that was more strongly worded than the strongest statement present in the associated journal article.

**Figure 2.**
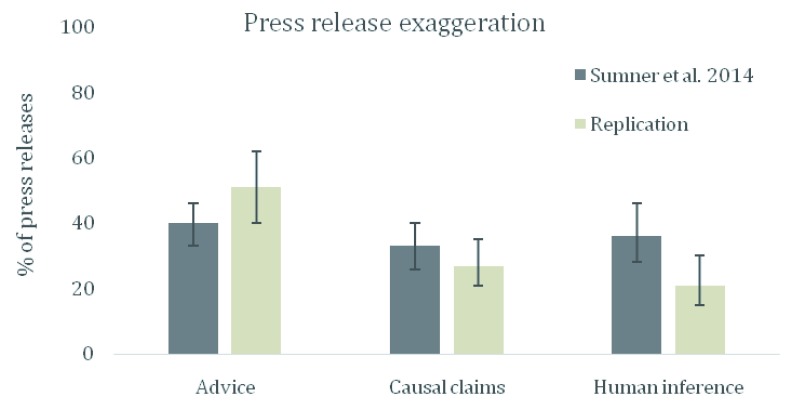
Percentage of press releases containing exaggeration compared to the journal article, for each of the three categories of exaggeration: advice, causal claims from correlational evidence and human inference from non-human samples (light grey bars). Data from the same analyses by Sumner *et al*. (2014) are presented for comparison (dark grey bars). Error bars are 95% confidence intervals.

***Human inference.*** In total, 21% (95% confidence interval = 15% to 30%) of press releases about non-human samples contained implicit or explicit references to human samples when the journal article did not. See Figure 2 for a comparison to Sumner *et al.* (2014).

### Association of news exaggeration with press releases

**Figure 3.**
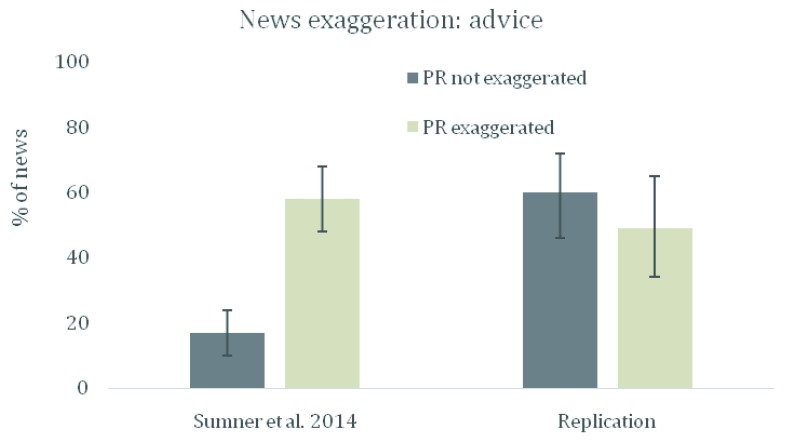
Percentage of news containing exaggerated advice when the press release was not exaggerated (dark grey bars) or exaggerated (light grey bars). The data for the same comparison by Sumner *et al*. (2014) are displayed for comparison.

***Advice.*** Overall, 55% (95% confidence interval = 44% to 65%) of news articles contained new advice, or a higher level of advice than the associated journal article. When press releases contained exaggerated advice, 49% (95% confidence interval = 34% to 65%) of the related news reports were also exaggerated in the same way. Conversely, when the level of advice in the press release was not in excess of that found in the journal article, 60% (95% confidence interval = 46% to 72%) of the associated news articles contained exaggerated advice. There was no significant relationship between exaggerated press releases and the presence of exaggerated advice in the news (difference = 11%, 95% confidence interval = -9.9% to 31.9%; odds ratio = 0.7, 95% confidence interval = 0.3 to 1.5). Figure 3 compares this outcome to the data from Sumner *et al.* (2014).

**Figure 4.**
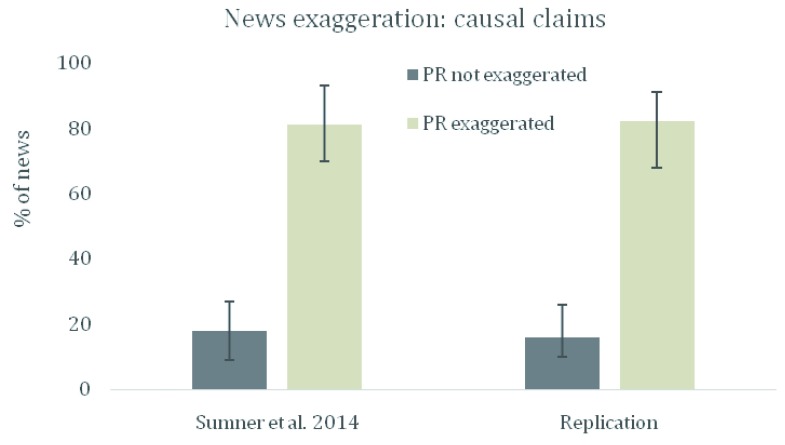
Percentage of news containing exaggerated causal claims from correlational evidence when the press release was not exaggerated (dark grey bars) or exaggerated (light grey bars). The data for the same comparison by Sumner *et al*. (2014) are displayed for comparison.

***Causal claims.*** The causal language used to describe correlational findings was stronger than that presented in the journal article in 49% (95% confidence interval = 37% to 61%) of news articles. When press releases contained exaggeration of causal language, 82% (95% confidence interval = 68% to 91%) of the associated news articles also contained exaggeration compared to 16% (95% confidence interval = 10% to 26%) of news articles when the press releases were not exaggerated. The difference between rates of exaggeration between conditions was 66% (95% confidence interval = 52.2% to 79.8%), and the odds of exaggeration in news were 23.7 times higher in relation to exaggerated press releases than representative press releases (odds ratio = 23.7, 95% confidence interval = 9.0 to 62.2). For a comparison of this data to the same analysis from Sumner *et al.* (2014), see Figure 4.

**Figure 5.**
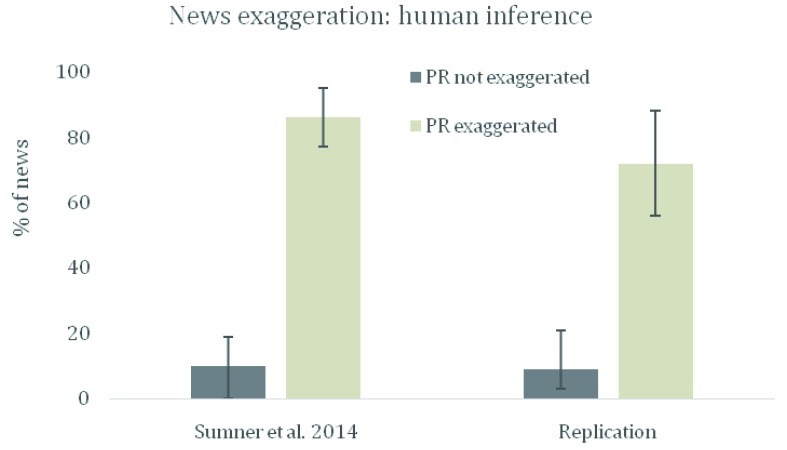
Percentage of news containing exaggerated human inference from non-human samples when the press release was not exaggerated (dark grey bars) or exaggerated (light grey bars). The data for the same comparison by Sumner *et al*. (2014) are displayed for comparison.

***Human inference from animal studies.*** For the comparison of reported samples, 33% of news articles included statements that made inferences relating to humans in excess of those present in the news articles. When press releases contained exaggerated statements, 72% (95% confidence interval = 46% to 88%) of the related news contained exaggeration, compared to 9% when the press release did not (95% confidence interval = 3% to 21%). The difference between conditions was 63% (95% confidence interval = 39.4% to 86.6%) and the odds of exaggeration in news were 26.5 times higher (95% confidence interval = 6.1 to 116.0). See Figure 5 for a comparison of this data to the same analysis by Sumner *et al.* (2014).

### Is news uptake associated with exaggeration in press releases?

There were no significant relationships between the presence of news coverage and exaggeration in press releases. For press releases with exaggerated advice, 57% (95% confidence interval = 39% to 74%) had associated news stories compared to 45% (95% confidence interval = 31% to 59%) for press releases without exaggerated advice (12% difference, 95% confidence interval = -11.5% to 35.5%). For press releases with exaggerated causal statements, 57% (95% confidence interval = 40% to 72%) had associated news articles compared to 56% (95% confidence interval = 44% to 66%) for press releases without exaggerated causal statements (1% difference, -18.6% to 20.6%). For press releases with exaggerated human inference from non-human samples, 44% (95% confidence interval = 27% to 62%) had associated news compared to 35% (95% confidence interval = 26% to 46%) for press releases without exaggerated human inference (9% difference, 95% confidence interval = -12.1% to 30.1%). Table 2 provides a comparison of the level of uptake for each analysis compared with the equivalent results from Sumner *et al.* (2014).

**Table 2.** Percentage of press releases with news stories comparing Sumner *et al*. (2014) with this replication.

Variable		Sumner *et al.* (2014)		Replication	
		Percentage with news	95% confidence interval	Percentage with news	95% confidence interval
Advice	Not exaggerated	52	43 - 60	45	31 - 59
	Exaggerated	59	48 - 69	57	39 - 74
	Difference	7	-6 - 21	12	-12 - 36
Language	Not exaggerated	50	41 - 60	56	46 - 66
	Exaggerated	57	44 - 69	57	40 - 72
	Difference	7	-9 - 22	1	-19 - 21
Sample	Not exaggerated	43	32 - 56	34	26 - 46
	Exaggerated	50	35 - 66	44	27 - 62
	Difference	7	-13 - 27	9	-12 - 30

## Discussion

The findings presented above are largely consistent with Sumner *et al*. (2014), with some notable exceptions. The similarities and differences are discussed in turn for each type of exaggeration.

### Advice

The main difference between the current results and the data of Sumner *et al.* (2014) is that here we found no significant association between exaggerated advice in news and press releases ( Figure 3). This may reflect the definition of exaggerated advice itself being insecure. Although some bodies recommend avoiding *de novo* advice (e.g. “Distinguish between findings and interpretation or extrapolation; don’t suggest health advice if none has been offered”, Science Media Centre, 2018), some press officers and journalists have expressed to us the view that it is sometimes their duty to include advice pertaining to the topic at hand. For example, where a study might find an association between chocolate and reduced risk of disease, many press officers and journalists would add advice not to over-indulge in chocolate in any case. In our study this scenario would have been coded as exaggeration if it was absent from the journal article, but it is not what the coding system was intended to capture (e.g. exaggerations such as advice to eat *more* chocolate). Relatedly, a change from advice not to the reader and advice to the reader was intended to capture instances of exaggeration such as ‘doctors should consider whether chocolate has a place in a patient’s healthy diet’ becoming ‘eat more chocolate’. However, there are many cases in which a change of readership (e.g. between journal articles, press releases and news) would justify a change in the wording of advice, which might in turn get coded as exaggeration. It is therefore very difficult to define one rule for whether advice is exaggerated, and we accept that our operationalization of advice will be problematic in some scenarios. For this reason, it may not be surprising that the pattern of results for advice can fluctuate across studies.

### Causal claims

The results for causal claims were highly similar to those in Sumner *et al.* (2014), showing similar levels of exaggeration, a similarly strong association between news and press releases, and no evidence for any difference in news uptake. However, it is worth noting that in replicating the analysis of Sumner *et al.* (2014), we used here a definition of exaggeration based on linguistic logic distinguishing the seven levels of relationship statement listed in Table 1. Subsequently, Adams *et al*. ( Adams *et al.*, 2017) offered a different definition based on reader understanding rather than linguistic logic. Adams *et al*. found that readers did not consistently distinguish between conditional, ambiguous and associative categories. Adams *et al*. (2017) reanalysed the causal claim data of Sumner *et al*. (2014) removing from the definition of exaggeration transitions between these categories. This reanalysis inevitably lowered absolute exaggeration rates but did not change the key results that news claims were strongly associated with press release claims, while exaggerations did not detectably raise news uptake. Convergently, a German translation of the seven-level scale was also condensed following pilot testing, removing the distinction between associative and ambiguous categories ( Buhse *et al.*, 2018). The exaggeration rate for news claims was similar to that reported here (45% of news headlines had stronger causal statements than the linked journal article). Since the results for causal claims appear to be robust both in replication and against different coding regimes, this represents a good basis for further research on causal claims in news ( Adams *et al*., 2019; Bott *et al*., 2019).

### Human inference from non-human samples

The human inference results also mainly replicated Sumner *et al.* (2014) in the strong association between news and press releases and the lack of evidence for changed news uptake. However, the overall rate of exaggeration was lower (outside the 95% CI of Sumner *et al.,* see Figure 2). We previously speculated that much of the exaggeration we observed across all categories was not intentional, but represented subtle changes in the message that occur for reasons other than deliberate hype. In the case of human inference, one motivation mentioned to us by researchers and press officers was to avoid identifying animal research facilities. The lower levels of human inference from non-human findings in 2014/15 compared to 2011 may be indicative of the success of the Concordat on Openness on Animal Research in the UK. The concordat was signed by the majority of the institutions in this replication from May 2014 onwards (i.e. during the sample period), and was developed from the Declaration of Openness on Animal Research from the end of 2012 by many of the sample institutions; that is, it was conceived after the publication of the press releases sampled from 2011 by Sumner *et al.* (2014), and before our sample in 2014. This concordat included the commitment that each signatory would “include information about that animal research in relevant communications, including media releases” (commitment 2 of the concordat, Concordat on Openness on Animal Research in the UK, 2014). It would seem likely that the concordat may therefore be responsible for an increase in explicitly mentioning animals in press releases, which would in turn reduce the proportion of claims expressed as if the study was on humans. This appears to be an example of the potential for positive outcome when institutions unite to address issues with science communication.

### Limitations

Limitations to Sumner *et al*. (2014) also apply to this replication. In the previous study, Sumner *et al.* (2014) described how the retrospective observational nature of the study design could not be used to make inferences about whether exaggeration in press releases causes exaggeration in news articles, and that same limitation is present here. Though it is clear that press releases are important sources of information for journalists, there are many sources of variation other than those reported in this study (for example: word count, study topic, reported statistics) which may play a role in news selection and content. There has been one experimental study in which identical sets of press releases differing only in the variables of interest were given to trainee journalists who assessed their newsworthiness (Experiment 4 in Bott *et al*. 2019). The press releases differed in two variables: strength of causal claim and presence/absence of a caveat about the causal claim. Neither factor appeared to influence the selection of newsworthy stories. These results are consistent with the lack of new uptake effects here. The trainee journalists were also asked to give reasons for their selections, and the most common reasons given were the appeal of the topic and the size of the audience.

Since the replication reported above examined articles from the same press offices and newsrooms as Sumner *et al*. (2014), it is a fairly direct, albeit narrow, replication. The sample of Dutch language articles used by Schat *et al*. (2018) represents a better opportunity to evaluate the robustness of the results of Sumner *et al*. (2014) in a wider European context, by authors entirely independent of the authors of the original study.

Although many of the important aspects of this replication are matched to the original study, one aspect where they differed was in the time frame of data collection. The original study selected all relevant press releases for the year 2011, but the data for our replication were collected for the periods of January to June in 2014 and 2015 (for a logistical reason not relevant to the analyses above). It is not clear whether there would be any difference in the selection of stories by the newsrooms, or whether news content would change from July onwards, but the output of press offices at the end of the year may be slower than at the start of the year. In the replication sample, there were 890 relevant press releases (before being restricted to a maximum of 20 per institution), in comparison to 462 relevant press releases in the original study. This increase could be an increase in output year-on-year, or it could be indicative of a differential within the year, which might possibly have some slight consequence for news uptake.

## Conclusion

The findings of this replication reinforce the main conclusions from Sumner *et al.* (2014), and broadly also those of Schat *et al.* (2018). Exaggerated claims in news are generally strongly associated with their presence in press releases, while there is little evidence that press release exaggeration attracts much more news. In the case of advice, the definition of exaggeration is open to debate and the results were not consistent with Sumner *et al.* (2014). The approach to coding and defining advice would need to be further developed for future research. In the case of causal claims, the results strongly mirrored previous data, and form a firm foundation for future research. In the case of non-human samples, the association between press release and news was mirrored, but the overall rate of exaggeration dropped, likely due to the Concordat on Openness on Animal Research in the UK (2014). Overall, these findings should be seen as further impetus for scientists and press officers “to communicate research accurately, without over-stating results and misleading the public – particularly when it comes to health” ( Burwell *et al.*, 2017). There appears to be no benefit to exaggerating results in terms of media coverage – only the danger that the public may be misinformed.

## Data availability

### Underlying data

Open Science Framework: ISRCTN10492618: RCT of optimal press release wording on health-related news coverage. https://doi.org/10.17605/OSF.IO/V8QHN ( Chambers *et al*., 2019).

Details of how to access and use the database can be found in file ‘Insciout_Documentation.html’. The underlying data used for this study can be found in folder ‘Raw data’, file ‘before_after_data; extracted data for this study can be found in folder ‘Processed data’, file ‘Replication_of_sumner2014_data’.

Data are available under the terms of the Creative Commons Attribution 4.0 International license (CC-BY 4.0).
