# Supplementary material for: The association between exaggeration in health-related science news and academic press releases: a replication study
Source: Wellcome Open Res. 2019 Nov 18;4:148. Originally published 2019 Oct 7. [Version 2] doi: 10.12688/wellcomeopenres.15486.2 (PMC6833989; doi:10.12688/wellcomeopenres.15486.2)
Supplement: Peer review history

# Peer review: Reviewer response for version 1

**DOI:** 10.21956/wellcomeopenres.16942.r36870

**Published:** 2019-11-05

**Authors:** Peter J. Schulz

**Affiliations:** 1 Institute of Communication and Health (ICH), University of Lugano, Lugano, Switzerland

This is a study on studies, more precisely on popularizing scientific research via the news media. Its subject is the issue of whether exaggeration in press releases about scientific studies pays. When faced with the necessity or a motivation to inform the public about our work, we are confronted with a predicament: do we act as a good PR operator or as a cautious researcher? The PR operator stresses, is selective, and must put complicated matters in short and memorable slogans; the researcher tends to complicate matters, has his caveats and knows the limits of his word. And in the core element of this research is concerned with is the exaggerated press release and its consequences for news coverage. The major result, in maybe exaggerated shortness, is that the exaggerations in scientific press releases issued by universities or journals is reflected in the news stories that appear in reaction to the press release, but the exaggerations do not enlarge the media reaction to research. In other word: if there are exaggerated press releases, the news media report differently, but they do not report more. Exaggerations do not increase public salience.

 The authors define conceptually as well as operationally three ways of exaggerating: unfounded advice, causal inference from correlational data, and conclusions about humans when no humans were studied. They manage to define variables and categories for such complicated phenomena and thus, as a side effect of their work, demonstrate the potentials for the content analysis method.

 The studies reported in this manuscript actually are precise replications of earlier and published articles up to minute methodological decisions. This activates in the reviewer the reaction to look for what is new in the research that you have before you. The answer to the question of what is new in this manuscript would be: nothing much. But a second thought reminds us of the value of replications, which are a much more frequent item on the research agenda of scientists as compared to the humanities. Replications are valuable because they help us to exclude the interpretation that the findings are a mere reflection of certain unalterable background features. Replications with small variety in background elements and stable or changed results help to attribute findings to background in some more realistic way. Therefore, we will not count the fact that this is a replication against indexing.

 However, the results mostly confirm the findings of similar earlier research and can now be regarded as pretty well sustained. This will open a possibility to include other indicators of the subjects of the study of press releases and their effect on news coverage on research. Aside from exaggeration, the abandoning of precision, the selection of technical terms, including some that have proven their utility in a scholarly discipline, might carry undesired elements into the public perception of sciencespeak and could have repercussions in the world of scientific inquiry proper. Therefore my advice is not to concentrate, in future work, on another replication, even if it were a better one, but rather widen the conceptual entities under scrutiny.

I confirm that I have read this submission and believe that I have an appropriate level of expertise to confirm that it is of an acceptable scientific standard.

# Response

**Published:** 2019-11-05

**Authors:** Petroc Sumner

**Affiliations:** Cardiff University, UK

Thank you for this review and for the good suggestions for future work. We agree that we would now expand the approach to further questions. We will put these specific ideas to our collaboration team for discussion. We are also branching in other ways: for example, we have been analysing conflicts of interest and research funding statements, and moving beyond press releases, we are pursuing a series of studies on how information campaigns affect flu vaccine attitudes and uptake (e.g. whether the myth-busting approach backfires)

# Peer review: Reviewer response for version 1

**DOI:** 10.21956/wellcomeopenres.16942.r36682

**Published:** 2019-10-25

**Authors:** David Colquhoun

**Affiliations:** 1 Department of Neuroscience, Physiology and Pharmacology, University College London, London, UK

It is great to see a replication study. It should certainly be indexed. The only suggestion that I have is that I think the statistical methods used should be described in much more detail. At present, it’s very brief.

 “Consistent with our previous approach ( Sumner *et al*., 2014), generalised estimating equations (GEE; with logit linking function and exchangeable working correlation; SPSS version 24) were used to provide estimates and confidence intervals adjusting for the clustering of multiple articles to one source (multiple news articles from one press release; or multiple press releases from the same institution)”.

 And the reference cited (Sumner *et al.* 2014) doesn’t give much more detail. 

 I think that all relevant equations should be provided, with commentary on what assumptions are made by the analysis.

I confirm that I have read this submission and believe that I have an appropriate level of expertise to confirm that it is of an acceptable scientific standard.

# Response

**Published:** 2019-10-25

**Authors:** Petroc Sumner

**Affiliations:** Cardiff University, UK

Thank you for providing this constructive review. We will provide more details for the statistical analysis in the revised version.
